# Detection of Colistin Resistance in *Salmonella enterica* Using MALDIxin Test on the Routine MALDI Biotyper Sirius Mass Spectrometer

**DOI:** 10.3389/fmicb.2020.01141

**Published:** 2020-06-03

**Authors:** Laurent Dortet, Rémy A. Bonnin, Simon Le Hello, Laetitia Fabre, Richard Bonnet, Markus Kostrzewa, Alain Filloux, Gerald Larrouy-Maumus

**Affiliations:** ^1^MRC Centre for Molecular Bacteriology and Infection, Department of Life Sciences, Faculty of Natural Sciences, Imperial College London, London, United Kingdom; ^2^Department of Bacteriology-Hygiene, Bicêtre Hospital, Assistance Publique – Hôpitaux de Paris, Le Kremlin-Bicêtre, France; ^3^EA7361 “Structure, Dynamic, Function and Expression of Broad Spectrum ß-Lactamases”, Paris-Sud University, LabEx Lermit, Faculty of Medecine, Le Kremlin-Bicêtre, France; ^4^French National Reference Centre for Antibiotic Resistance, Le Kremlin-Bicêtre, France; ^5^Institut Pasteur, French National Reference Centre for E. coli, Shigella and Salmonella, Paris, France; ^6^Department of Bacteriology, University Hospital of Clermont-Ferrand, Clermont-Ferrand, France; ^7^Bruker Daltonik GmbH, Bremen, Germany

**Keywords:** MALDI mass spectrometry, lipid A, colistin, *Salmonella enterica*, diagno stic

## Abstract

Resistance to polymyxins in most Gram-negative bacteria arises from chemical modifications to the lipid A portion of their lipopolysaccharide (LPS) mediated by chromosomally encoded mutations or the recently discovered plasmid-encoded *mcr* genes that have further complicated the landscape of colistin resistance. Currently, minimal inhibitory concentration (MIC) determination by broth microdilution, the gold standard for the detection of polymyxin resistance, is time consuming (24 h) and challenging to perform in clinical and veterinary laboratories. Here we present the use of the MALDIxin to detect colistin resistant *Salmonella enterica* using the MALDxin test on the routine matrix-assisted laser desorption ionization (MALDI) Biotyper Sirius system.

## Introduction

Due to the limited pipeline of new antibiotics the increasing trend in antibiotic resistance is now threatening the global health. One of the most fearing issues is the dissemination of multidrug resistance (MDR) in Gram-negative bacteria. Indeed, these MDR bacteria may accumulate in a single strain resistance to the main classes of antimicrobial molecules, making colistin one of the last resort therapies for the treatment of infection caused by MDR Gram-negative bacteria.

In Gram-negative bacteria, acquired resistance to colistin results mostly from modifications of the drug target, i.e., the lipopolysaccharide (LPS). These modifications correspond to addition(s) of cationic groups such as 4-amino-L-arabinose (L-Ara4N) and/or phosphoethanolamine (pETN) on the lipid A, the anchor of the LPS (Olaitan et al., 2014; Zhang et al., 2019b). These modifications may result from chromosome-encoded mechanisms such as modification (basically mutations) of the two-component systems PmrA/PmrB and PhoP/PhoQ or alteration (mutation, disruption, down-regulation) of the global regulator MgrB. These chromosome-encoded mechanisms are mostly associated with addition of L-Ara4N on the lipid A. It has been reported that addition of pETN to lipid A can also occur through the expression of a plasmid-encoded pETN transferase, named mobilized colistin resistance (MCR) (Chew et al., 2017; Jayol et al., 2017; Jeannot et al., 2017;

Poirel et al., 2017; Zhang et al., 2019a). Despite, *mcr* genes have been described in many different enterobacteriales species, they are more prevalent in *Escherichia coli* and *Salmonella enterica* than in other Enterobacterales (Poirel et al., 2017). Of note, MCR-producing *S. enterica* have been often reported in livestock animals, mostly in chicken in which they are part of the normal gut flora. Overall, it is now accepted that the dissemination of *mcr* genes in animals was mostly driven by the intensive use of polymyxins during decades in livestock farming. With the soaring descriptions of MCR-producing isolates in animals during the 2 years following the original report of this mechanism, the use of colistin in veterinary medicine has been restricted in Europe (Catry et al., 2015). In addition, recommandations have been made for the reinforcement of the systematic monitoring of bacteria from food-producing animals for resistance to colistin (polymyxins). In 2013, monitoring and reporting of colistin resistance in commensal and zoonotic bacteria isolated from samples from certain food-producing animal populations and certain food became mandatory in Europe (European Union Commission, 2013). Accordingly, rapid detection of colistin resistance is therefore one of the key issues, not only in the human medicine to improve the treatment of patient infected with MDR bacteria, but also in the veterinary medicine where high-throughput methods would be of utmost interest. Unfortunately, detection of colistin resistance still relies on the determination of colistin susceptibility by performing minimal inhibitory concentration (MIC) using broth microdilution with 24 h of incubation time. Indeed, this susceptibility testing method remains the unique gold standard to identify colistin resistance. Therefore, there is an urgent need to develop a fast and robust assay to detect colistin-resistant Gram-negative bacteria including *S. enterica* (Carroll et al., 2019; Lima et al., 2019). Ultimately, this test should be a high-throughput assay to manage a large number of samples as it is the case in food industry.

Recently, we developed a rapid technique using matrix-assisted laser desorption ionization time-of-flight (MALDI-TOF) able to detect colistin resistance directly from whole bacteria in less than 15 min, the MALDIxin test (Dortet et al., 2018a, b). Here, we report on the application of the optimized MALDIxin, which has been applied to *E. coli*, *K. pneumoniae* and *Acinetobacter baumannii* (Dortet et al., 2018a, b, 2019; Furniss et al., 2019), using routine MALDI mass spectrometer and its application into the detection of colistin-resistant *S. enterica*.

## Materials and Methods

### Bacterial Strains

A collection of 23 *S. enterica* clinical strains was used, which included 12 colistin resistant isolates and 11 colistin susceptible isolates. Among the resistant isolates, 8 were MCR producers: 6 were MCR-1, 1 was MCR-4 and 1 was MCR-5 (Table 1). All the strains used in this study were part of the strain collections from the French National Reference Centre (NRC) for Antimicrobial Resistance, where all patient information are very partial and anonymized. The strains were not isolated for the purpose of our study, so ethical approval and consent were not required.

**TABLE 1:** GenBank accession number of colistin resistant and colistin susceptible *Salmonella enterica* isolates used in this study.

**Strain**	**Name**	**Serotype**	**GenBank accession number**	**Colistin MIC (mg/L)**	**colistin resistance mechanism**	**Ref.**
**Colistin resistant strains**						
Sal-R1	201607059	4,12:i:- (monophasic)	SAMN13531479	4	*mcr-1*	This study
Sal-R2	201606765	4,12:i:- (monophasic)	SAMN13531480	8	*mcr-1*	This study
Sal-R3	201609932	4,5,12:i:- (monophasic)	SAMN13531481	8	*mcr-1*	This study
Sal-R4	201610655	4,12:i:- (monophasic)	SAMN13531482	8	*mcr-1*	This study
Sal-R5	201610686	Paratyphi B d-tartrate + (biotype Java)	SAMN13531483	8	*mcr-1*	This study
Sal-R6	CNR 1776	Typhimurium	JAAOHZ000000000	8	*mcr-1*	This study
Sal-R7	13-SA01718	Paratyphi B d-tartrate + (biotype Java)	PRJNA396070	8	*mcr-5*	This study
Sal-R8	201600129	Dublin	SAMN13531484	4	Unknown	This study
Sal-R9	201607119	Enteritidis	SAMN13531485	4	mutated MgrB (K3T)	This study
Sal-R10	201606219	Typhimurium	SAMN13531486	4	mutated MgrB (Q30R)	This study
Sal-R11	201600169	Enteritidis	SAMN13531487	4	Unknown	This study
Sal-R12	R3445	4,12:i:- (monophasic)	MF543359	8	*mcr-4*	This study
**Colistin susceptible strains**						
Sal-S2	201604739	4,12:i:- (monophasic)	SAMN13531488	1	–	This study
Sal-S3	201604769	Enteritidis	SAMN13531489	2	–	This study
Sal-S4	201605339	4,12:i:- (monophasic)	SAMN13531490	1	–	This study
Sal-S5	201608919	Enteritidis	SAMN13531491	1	–	This study
Sal-S6	201606509	Typhimurium	SAMN13531492	1	–	This study
Sal-S7	201602769	Anatum	SAMN13531493	1	–	This study
Sal-S8	201606129	4,12:i:- (monophasic)	SAMN13531494	2	–	This study
Sal-S9	201607559	Enteritidis	SAMN13531495	0.5	–	This study
Sal-S10	201606439	4,12:i:- (monophasic)	SAMN13531496	1	–	This study
Sal-S11	201610299	Veneziana	SAMN13531497	0.5	–	This study
Sal-S12	201606239	Chester	SAMN13531498	2	–	This study

### Susceptibility Testing

Minimal inhibitory concentrations were determined by broth microdilution (BMD) according to the guidelines of the Clinical and Laboratory Standards Institute (CLSI) and European Committee on Antimicrobial Susceptibility Testing (EUCAST) joint subcommittee. Results were interpreted using EUCAST breakpoint as updated in 2019 (EUCAST, 2019).

### Whole Genome Sequencing (WGS)

Whole Genome Sequencing was performed by the “Plateforme de Microbiologie Mutualisée (P2M)” at Institut Pasteur (Paris, France) for all colistin resistant isolates. Briefly, total DNA was isolated using the MagnaPure^®^ microbial DNA isolation kit (Roche Laboratories) from overnight cultures on Mueller-Hinton agar (BioRad, Marnes-la-Coquette, France). Genomic DNA quantifications were performed using Qubit fluorometer (Life Technologies, Carlsbad, CA) and adjusted at 0.2 ng/μl. Library preparation was performed using NextEra^®^ XT DNA sample preparation kit (Illumina, San Diego, CA, United States). Sequencing was performed on an Illumina NextSeq500 sequencer with v3 chemistry using 2×150-bp paired-end reads at a raw cluster density of~1300,000 clusters/mm^2^. Paired-end reads varied in read length depending on the sequencing platform/site, from 100 to 146 bp, yielding a minimum of 30-fold coverage per isolate.

### Bioinformatic Analysis

Raw data were assembled into contigs using CLC Genomics Workbench v9.5.3^1^. To identify *mcr* genes, total raw data sequences of each isolate were subjected to ResFinder-2.1 Server^2^ that is dedicated to the identification of acquired antimicrobial resistance genes. To identify mutation in PmrA, PmrB, PhoP, PhoQ and the master regulator MgrB (Supplementary Figure S2), sequences alignments were performed using ClustalW^3^. Sequence of *Salmonella enterica* subsp. *enterica* serovar Newport str. USMARC-S3124.1 (GenBank accession number CP006631) was used as reference sequence.

### Nucleotide Sequence Accession Number

The whole genome sequences generated in the study have been submitted to the GenBank nucleotide sequence database under the accession number detailed in Table 1.

### MALDIxin Test

A 10 μL inoculation loop of bacteria, grown on Mueller-Hinton agar for 18–24 h, was resuspended in 200 μL of water. Mild-acid hydrolysis was performed on 100 μL of this suspension, by adding 100 μl of acetic acid 2 % v/v and incubating the mixture at 98°C for 10 min. Hydrolyzed cells were centrifuged at 17,000 ×*g* for 2 min, the supernatant was discarded and the pellet was resuspended in ultrapure water to a density of McFarland 10. A volume of 0.4 μL of this suspension was loaded onto the MALDI target plate and immediately overlaid with 1.2 μL of a matrix consisting of a 9:1 mixture of 2,5-dihydroxybenzoic acid and 2-hydroxy-5-methoxybenzoic acid (super-DHB, Sigma-Aldrich) solubilized in chloroform/methanol 90:10 v/v to a final concentration of 10 mg/mL.

The bacterial suspension and matrix were mixed directly on the target by pipetting and the mix dried gently under a stream of air. MALDI-TOF mass spectrometry analyses were performed with a MALDI Biotyper Sirius (Bruker Daltonics) using the linear negative-ion mode.

### Data Analysis

The negative mass spectrum was scanned between *m*/*z* 1,600 and *m*/*z* 2,200 in the negative linear ion mode. Manual peak picking at masses relevant to colistin resistance was performed on the obtained mass spectra and the corresponding signal intensities at these defined masses was determined. The percentage of modified lipid A was calculated by dividing the sum of the intensities of the lipid A peaks attributed to addition of pETN (*m/z* 1919.2 and *m/z* 2157.2) and L-Ara4N (*m/z* 1927.2 and *m/z* 2165.2) by the intensity of the peaks corresponding to native lipid A (*m/z* 1796.2 and *m/z* 2034.2). All mass spectra were generated and analyzed in technical triplicate (i.e., measurements of each sample were repeated three times) and biological triplicate.

### Statistical Analysis

All experiments were carried out on three independent bacterial cultures. Data were compared two-by-two using unpaired Mann-Whitney test. *P* values <0.05 were considered statistically different using GraphPad Prism 7.

## Results and Discussion

**FIGURE 1:**
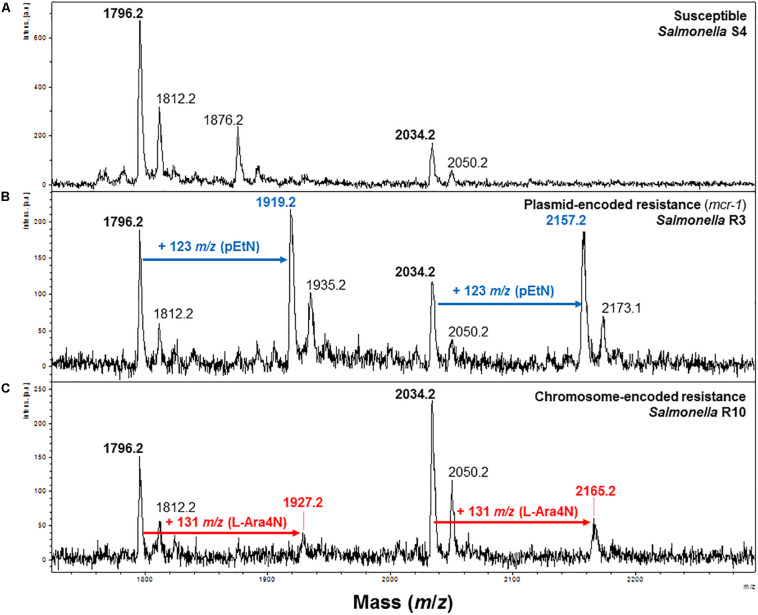
Representative mass spectra of susceptible and modified *S. enterica* lipid A acquired using the linear negative-ion mode of a MALDI Biotyper Sirius system (Bruker Daltonics). **(A)** Susceptible *S. enterica* lipid A is detected as two major peaks at *m/z* 1796.2 and *m*/*z* 2034.2 (in bold). **(B)** Lipid A from *S. enterica* strains exhibiting MCR-mediated resistance to colistin exhibit additional peak at *m/z* 1919.2 and *m/z* 2157.2 (in blue) corresponding to pETN addition on the natural lipid A (peaks at *m/z* 1796.2 and *m*/*z* 2034.2). **(C)** Lipid A from colistin-resistant *S. enterica* isolates carrying chromosomal mutations is modified by L-Ara4N which are detected as additional peaks at *m/z* 1927.2 and *m/z* 2165.22 (in red).

As shown in Figure 1A and Supplementary Figure S1, the mass spectrum of colistin susceptible *S. enterica* is dominated by a set of three of peaks assigned to bis-phosphorylated hexa-acyl lipid A, tri-phosphorylated hexa-acyl lipid A and bis-phosphorylated hepta-acyl lipid A. The major peaks at *m*/*z* 1796.2 and *m*/*z* 1876.2 correspond to hexa-acyl diphosphoryl and hexa-acyl triphosphoryl lipid A, respectively, containing four C14:0 3-OH, one C14:0 and one C12:0. The peak at *m*/*z* 2034.2 corresponds to hepta-acyl diphosphoryl lipid A four C14:0 3-OH, one C14:0, one C12:0 and one C16:0. Peaks at *m*/*z* 1812.2 and *m*/*z* 2050.2 are also observed in colistin susceptible *Salmonella enterica* and which can be tentatively be assigned to hexa-acyl diphosphoryl lipid A, containing five C14:0 3-OH and one C12:0, and hepta-acyl diphosphoryl lipid A five C14:0 3-OH, one C12:0 and one C16:0, respectively.

**FIGURE 2:**
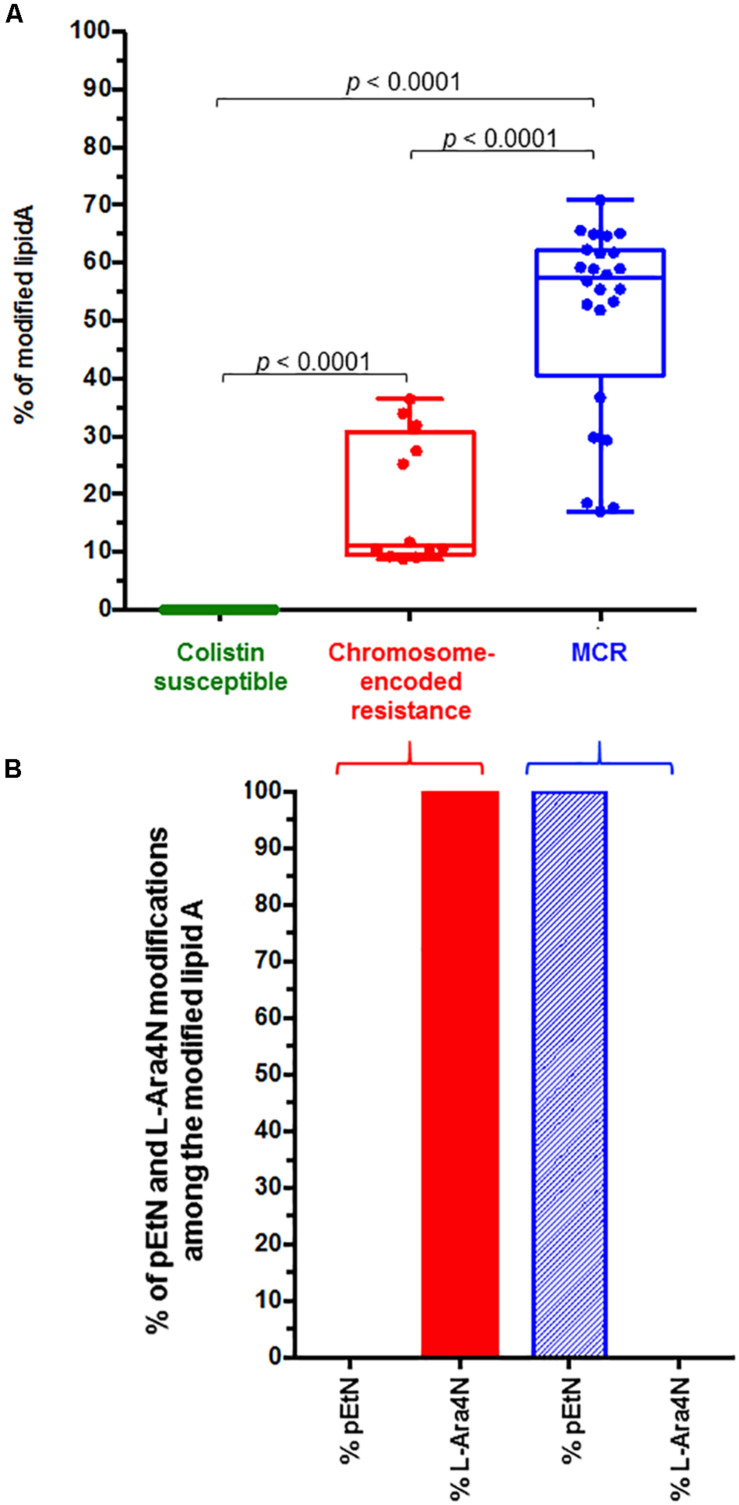
**(A)** Representation of the percentage of the modified lipid A for colistin susceptible and colistin resistant *S. enterica* isolates. The global percentage of modified lipid A (L-Ara4N + pETN modified lipid A / native lipid A) is represented for colistin susceptible strains (*n* = 11), colistin chromosome-encoded resistant *S. enterica* isolates (*n* = 4) and MCR-producing *S. enterica* isolates (*n* = 8). All experiments were performed in triplicate. **(B)** Representation of the percentage of L-Ara4N and pETN modified lipid A among the global modified lipid A for colistin resistant *S. enterica* isolates.

In MCR-producing *S. enterica* strains additional peaks at *m*/*z* 1919.2, *m*/*z* 1935.2, *m*/*z* 2157.2 and *m*/*z* 2173.2 were observed (Figure 1B). Those peaks correspond to the addition of one phosphoethanolamine (pETN) moiety to the phosphate group at position 1 of the native lipid A, leading to an increase of +123 mass units compared to the mass of the major peak of native lipid A at *m*/*z* 1796.2, *m*/*z* 1812.2, *m*/*z* 2034.2 and *m*/*z* 2050.2. In *mcr* negative colistin-resistant *S. enterica* strains, two additional peaks at *m/z* 1927.2 and *m/z* 2165.2 were observed. These signals correspond to the addition of 4-amino-L-arabinose (L-Ara4N) to the 4’-phosphate of the native lipid A, resulting in an increase of +131 *m/z* compared to the native lipid A peak at *m/z* 1927.2 and *m*/*z* 2034.2 (Figure 1C). As shown in Figure 2A and Table 1, the average percentage of modified lipid A remained significantly lower in chromosome-encoded colistin resistant isolates (18.25 ± 3.23%) compared to MCR-producers (51.12 ± 3.38%) (data not shown). The same observation was previously reported with *Klebsiella pneumoniae* and *E. coli* (Dortet et al., 2019; EUCAST, 2019). As expected, only pETN modification of the lipid A was observed in MCR-producers whereas only L-Ara4N addition could be detected in all chromosome-encoded colistin-resistant strains (Figure 2B and Table 1). Again, these results are in perfect agreement with those obtained in *K. pneumoniae* and *E. coli*.

Overall, the MALDIxin test is able to detect colistin resistance in *S. enterica* and to discriminate between chromosome-encoded resistance and MCR-producers. By targeting the modification of native lipid A, especially pETN addition, MALDIxin test is able to detect all variants of MCR. This is of the utmost importance since the increasing number of *mcr* variants (*mcr-1* to *mcr-9*, as of today) do not allow to perform a simple test (e.g., PCR) to detect all *mcr* variants. In addition, in some rare case, the *mcr* gene is not expressed, leading to colistin susceptibility but positive PCR, including in *S. enterica* isolates (Carroll et al., 2019). Finally, the MALDIXin test possesses high-throughput capacities that might be crucial in the context of large-scale screening. Accordingly, this test might be useful to answer the unmet need of a rapid, robust and high-throughput test for identification of colistin resistance that exist in veterinary medicine and food industry, where monitoring of colistin resistance in *S. enterica* is mandatory. However, one limitation of this study is the sample size used which might represent a fraction of the different types of colistin resistance mechanisms in *S. enterica*.

## Conclusion

The MALDIxin test is now optimized on a routine machine, the MALDI Biotyper Sirius, for the detection of colistin resistance in *E. coli*, *K. pneumoniae*, *Acinetobacter baumannii*, and *S. enterica* (Dortet et al., 2018a, b, 2019; Furniss et al., 2019). The future development of automated algorithm and dedicated consumables (e.g., calibration standards, pre-portioned purified matrix) will also be able to standardize and simplify the assay, allowing its use in clinical and veterinary laboratories.

## Data Availability Statement

All datasets generated for this study are included in the article/Supplementary Material.

## Author Contributions

GL-M and LD conceived the study, participated in its design and preformed the experiments. SL provided clinical isolates. LD, RB, SL, MK, AF, and GL-M wrote the manuscript. All authors discussed, reviewed, and approved the final manuscript.

## Conflict of Interest

LD, AF, and GL-M are co-inventors of the MALDIxin test for which a patent has been filed by Imperial Innovations. MK is employee of Bruker, the manufacturer of the MALDI-TOF MS used in this study. The remaining authors declare that the research was conducted in the absence of any commercial or financial relationships that could be construed as a potential conflict of interest.

## Footnotes

1 www.clcbio.com

2 http://www.genomicepidemiology.org/

3 https://www.genome.jp/tools-bin/clustalw
