# Supplementary material for: Detection of Colistin Resistance in Salmonella enterica Using MALDIxin Test on the Routine MALDI Biotyper Sirius Mass Spectrometer
Source: Front Microbiol. 2020 Jun 3;11:1141. doi: 10.3389/fmicb.2020.01141 (PMC7283459; doi:10.3389/fmicb.2020.01141)
Supplement: Supplementary file 1 [file Table_1.docx]

**Table S1.** Genbank accession number of colistin resistant and colistin susceptible *Salmonella enterica* isolates used in this study

| **Strain** | **Name** | | **Serotype** | | **GenBank accession number** | | **Colistin MIC (mg/L)** | | **colistin resistance mechanism** | | **Ref.** | |  |
| --- | --- | --- | --- | --- | --- | --- | --- | --- | --- | --- | --- | --- | --- |
| **Colistin resistant strains** | | | | | | | | | | | | | |
| Sal-R1 | 201607059 | 4,12:i:- (monophasic) | | SAMN13531479 | | 4 | | *mcr-1* | | This study | |  |  |
| Sal-R2 | 201606765 | 4,12:i:- (monophasic) | | SAMN13531480 | | 8 | | *mcr-1* | | This study | |  |  |
| Sal-R3 | 201609932 | 4,5,12:i:- (monophasic) | | SAMN13531481 | | 8 | | *mcr-1* | | This study | |  |  |
| Sal-R4 | 201610655 | 4,12:i:- (monophasic) | | SAMN13531482 | | 8 | | *mcr-1* | | This study | |  |  |
| Sal-R5 | 201610686 | Paratyphi B d-tartrate + (biotype Java) | | SAMN13531483 | | 8 | | *mcr-1* | | This study | |  |  |
| Sal-R6 | CNR 1776 | Typhimurium | | JAAOHZ000000000 | | 8 | | *mcr-1* | | This study | |  |  |
| Sal-R7 | 13-SA01718 | Paratyphi B d-tartrate + (biotype Java) | | PRJNA396070 | | 8 | | *mcr-5* | | (17) | |  |  |
| Sal-R8 | 201600129 | Dublin | | SAMN13531484 | | 4 | | Unknown | | This study | |  |  |
| Sal-R9 | 201607119 | Enteritidis | | SAMN13531485 | | 4 | | mutated MgrB (K3T) | | This study | |  |  |
| Sal-R10 | 201606219 | Typhimurium | | SAMN13531486 | | 4 | | mutated MgrB (Q30R) | | This study | |  |  |
| Sal-R11 | 201600169 | Enteritidis | | SAMN13531487 | | 4 | | Unknown | | This study | |  |  |
| Sal-R12 | R3445 | 4,12:i:- (monophasic) | | MF543359 | | 8 | | *mcr-4* | | (18) | |  |  |
| **Colistin susceptible strains** | | | | | | | | | | | | | |
| Sal-S2 | 201604739 | | 4,12:i:- (monophasic) | | SAMN13531488 | | 1 | | - | | This study | |  |
| Sal-S3 | 201604769 | | Enteritidis | | SAMN13531489 | | 2 | | - | | This study | |  |
| Sal-S4 | 201605339 | | 4,12:i:- (monophasic) | | SAMN13531490 | | 1 | | - | | This study | |  |
| Sal-S5 | 201608919 | | Enteritidis | | SAMN13531491 | | 1 | | - | | This study | |  |
| Sal-S6 | 201606509 | | Typhimurium | | SAMN13531492 | | 1 | | - | | This study | |  |
| Sal-S7 | 201602769 | | Anatum | | SAMN13531493 | | 1 | | - | | This study | |  |
| Sal-S8 | 201606129 | | 4,12:i:- (monophasic) | | SAMN13531494 | | 2 | | - | | This study | |  |
| Sal-S9 | 201607559 | | Enteritidis | | SAMN13531495 | | 0.5 | | - | | This study | |  |
| Sal-S10 | 201606439 | | 4,12:i:- (monophasic) | | SAMN13531496 | | 1 | | - | | This study | |  |
| Sal-S11 | 201610299 | | Veneziana | | SAMN13531497 | | 0.5 | | - | | This study | |  |
| Sal-S12 | 201606239 | | Chester | | SAMN13531498 | | 2 | | - | | This study | |  |
