# Supplementary material for: Detection of Colistin Resistance in Salmonella enterica Using MALDIxin Test on the Routine MALDI Biotyper Sirius Mass Spectrometer
Source: Front Microbiol. 2020 Jun 3;11:1141. doi: 10.3389/fmicb.2020.01141 (PMC7283459; doi:10.3389/fmicb.2020.01141)

**Detection of colistin resistance in *Salmonella enterica* using MALDIxin test on the routine MALDI Biotyper Sirius mass spectrometer**

Laurent DORTET^1,2,3,4^, Rémy A. BONNIN^2,3,4^, Simon LE HELLO^5^, Laetitia FABRE^5^, Richard BONNET^4,6^, Markus KOSTRZEWA^7^, Alain FILLOUX^1^ and Gerald LARROUY-MAUMUS^1*^

^1^MRC Centre for Molecular Bacteriology and Infection, Department of Life Sciences, Faculty of Natural Sciences, Imperial College London, London, SW7 2AZ, UK

^2^Department of Bacteriology- Hygiene, Bicêtre Hospital, Assistance Publique - Hôpitaux de Paris, Le Kremlin-Bicêtre, France

^3^EA7361 “Structure, dynamic, function and expression of broad spectrum β-lactamases”, Paris-Sud University, LabEx Lermit, Faculty of Medecine, Le Kremlin-Bicêtre, France

^4^ French National Reference Centre for Antibiotic Resistance, France

^5^ Institut Pasteur, French National Reference Centre for *E. coli*, *Shigella* and *Salmonella*, France

^6^Bacteriology unit, University hospital of Clermont-Ferrand, Clermont-Ferrand, France

^7^Bruker Daltonik GmbH, Bremen, Germany

**Figure S1**: Mass spectra of the susceptible *S. enterica* lipid A acquired using the linear negative-ion mode of a MALDI Biotyper Sirius system (Bruker Daltonics).


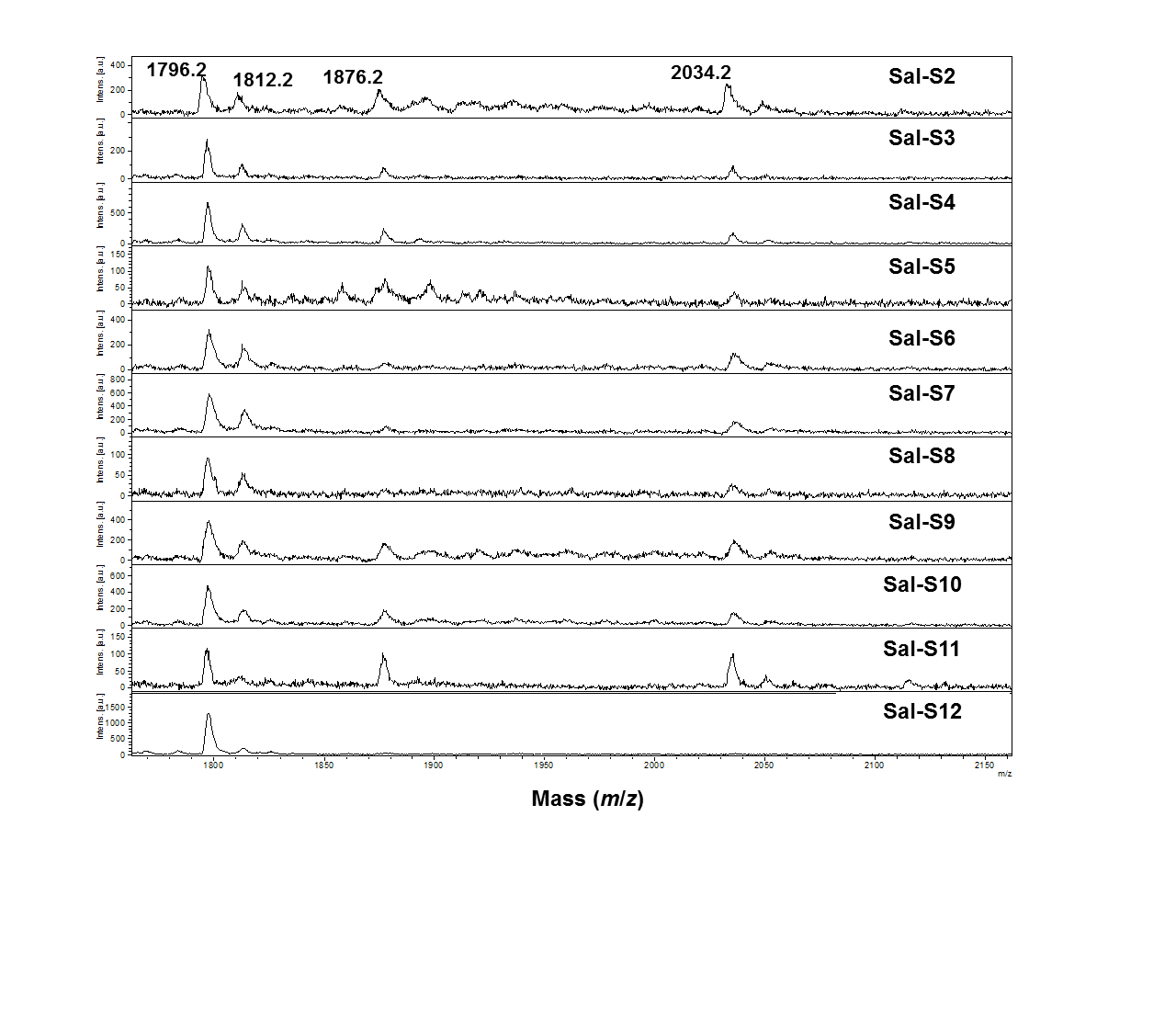


**Figure S2**: Sequence alignment of MgrB protein performed using ClustalW


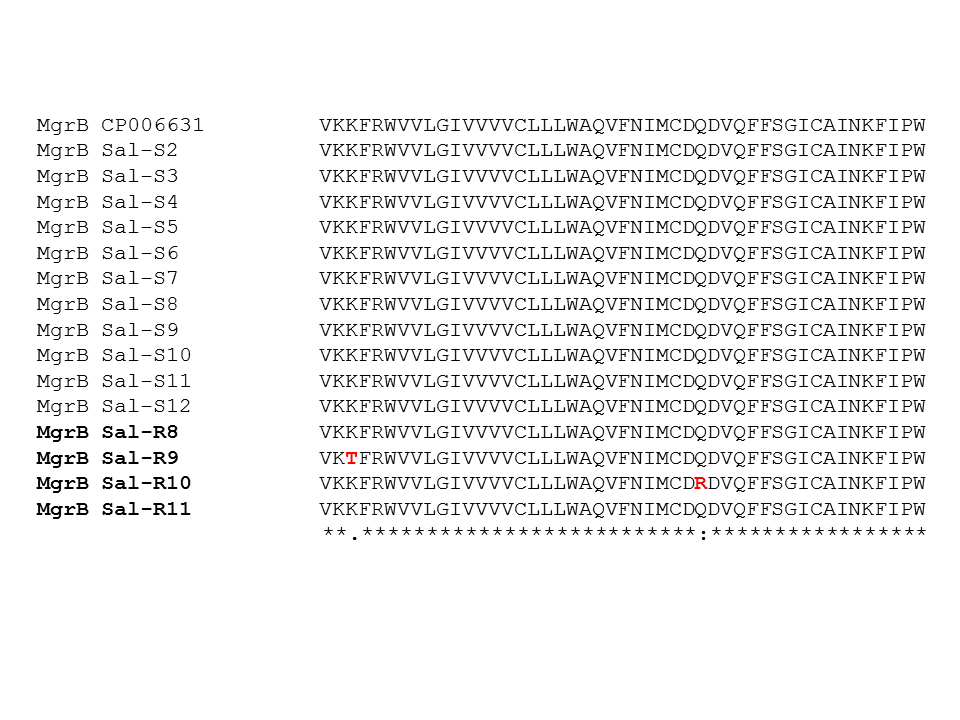

Supplement: Supplementary file 2 [file Data_Sheet_1.docx]
